# Integrative Bioinformatic Analysis of Transcriptomic Data Identifies Conserved Molecular Pathways Underlying Ionizing Radiation-Induced Bystander Effects (RIBE)

**DOI:** 10.3390/cancers9120160

**Published:** 2017-11-25

**Authors:** Constantinos Yeles, Efstathios-Iason Vlachavas, Olga Papadodima, Eleftherios Pilalis, Constantinos E. Vorgias, Alexandros G. Georgakilas, Aristotelis Chatziioannou

**Affiliations:** 1 Department of Biochemistry and Molecular Biology, Faculty of Biology, National and Kapodistrian University of Athens, Zografou Campus, 15701 Athens, Greece; yeles.konstantinos@gmail.com (C.Y.); cvorgias@biol.uoa.gr (C.E.V.); 2 Metabolic Engineering and Bioinformatics Research Team, Institute of Biology Medicinal Chemistry & Biotechnology, National Hellenic Research Foundation, 11635 Athens, Greece; svlachavas@eie.gr (E.-I.V); opapadod@eie.gr (O.P.); 3 Department of Molecular Biology and Genetics, Democritus University of Thrace, 68100 Dragana, Greece; 4 Enios Applications Private Limited Company, A17671 Athens, Greece; epilalis@eie.gr; 5 Physics Department, School of Applied Mathematical and Physical Sciences, National Technical University of Athens (NTUA), Zografou, 15780 Athens, Greece; alexg@mail.ntua.gr

**Keywords:** bioinformatics, ionizing radiation, microarrays, radiation-induced bystander effects, transcriptomics

## Abstract

Ionizing radiation-induced bystander effects (RIBE) encompass a number of effects with potential for a plethora of damages in adjacent non-irradiated tissue. The cascade of molecular events is initiated in response to the exposure to ionizing radiation (IR), something that may occur during diagnostic or therapeutic medical applications. In order to better investigate these complex response mechanisms, we employed a unified framework integrating statistical microarray analysis, signal normalization, and translational bioinformatics functional analysis techniques. This approach was applied to several microarray datasets from Gene Expression Omnibus (GEO) related to RIBE. The analysis produced lists of differentially expressed genes, contrasting bystander and irradiated samples versus sham-irradiated controls. Furthermore, comparative molecular analysis through BioInfoMiner, which integrates advanced statistical enrichment and prioritization methodologies, revealed discrete biological processes, at the cellular level. For example, the negative regulation of growth, cellular response to Zn^2+^-Cd^2+^, and Wnt and NIK/NF-kappaB signaling, thus refining the description of the phenotypic landscape of RIBE. Our results provide a more solid understanding of RIBE cell-specific response patterns, especially in the case of high-LET radiations, like α-particles and carbon-ions.

## 1. Introduction

Over the past years, novel approaches in radiation biology and therapy have emphasized the importance of the study of systemic phenomena that represent non-targeted [1] radiation-induced bystander effects (RIBE) [2].

In detail, ionizing radiation (IR) damages the cellular genome directly or indirectly through the generation of reactive oxygen and nitrogen species (ROS/RNS) [3,4]. Undoubtedly, it has been demonstrated in various in-vitro and in-vivo studies that targeted irradiation of cytoplasm with α-particles IR induces mutations in the genome of the irradiated cells [5]. In this phenomenon, non-irradiated cells, adjacent to the irradiated cells, namely bystander cells, manifest stress responses as a result of signals derived from adjacent directly irradiated cells [6]. In addition, it has been illustrated that RIBE are linked to distinct molecular mechanisms, such as cell growth [7], micronuclei formation [8], cell cycle delay [7,9], and repair [5], along with the transformation of non-irradiated cells [10], inflammation, and DNA damage [5]. Recently, various “omics”-technologies (microarrays, Next Generation Sequencing (NGS)) have generated numerous transcriptomic datasets for the interrogation of the systemic character of the above phenomena.

Exploiting this fact, we analyzed various publicly available microarray datasets retrieved from Gene Expression Omnibus (GEO) [11,12], which is a repository that archives, annotates, and freely shares high-throughput functional genomics data submitted by the international research community. Our aim was to reveal the crucial molecular pathways, consistently involved in RIBE biology, responsible for different phenotypic features. We screened for common and different biological processes characterizing directly irradiated and bystander cells for low- and high-LET radiations, like α-particles and carbons. In particular, utilizing the BioInfoMiner [13] interpretation web platform, we were able not only to identify overrepresented functional terms, but also to pinpoint subsets of genes with pivotal roles in RIBE. BioInfoMiner is used here to perform functional enrichment analyses and gene prioritization for lists of differentially expressed genes, derived from statistical tests applied to the data of the examined microarray datasets. Through exploitation of the topological structure of the ontological trees it analyzes, BioInfoMiner automatically corrects these graphs for semantic inconsistencies they may include and derives a subset of the input genes, with pivotal genes, ranked according to their functional association with multiple, distinct, cellular processes. These genes, termed "linker genes", represent critical players in various distinct biological processes, enabling a systemic perspective of the disease under investigation Moreover, we demonstrated that the modularity of the RIBE systemic response elicits differentiated biological responses according to the particular type of radiation, while operating through conserved biological circuits, which exert their effect through common differentially expressed genes, such as *IL1A, IL1B, NFKBIZ, SAT1, and TNFAIP3,* in the majority of the datasets.

## 2. Results

### 2.1. Statistical Inference and Differential Expression

In order to decipher any differential expression patterns induced by RIBE, we applied a generic, proprietary computational workflow to each dataset separately (see Materials and Methods). The main statistical comparisons of interest concerned bystander vs. control and irradiated vs. control samples. Firstly, the differential expression results of all datasets are illustrated in Table 1.

**Figure 1:**
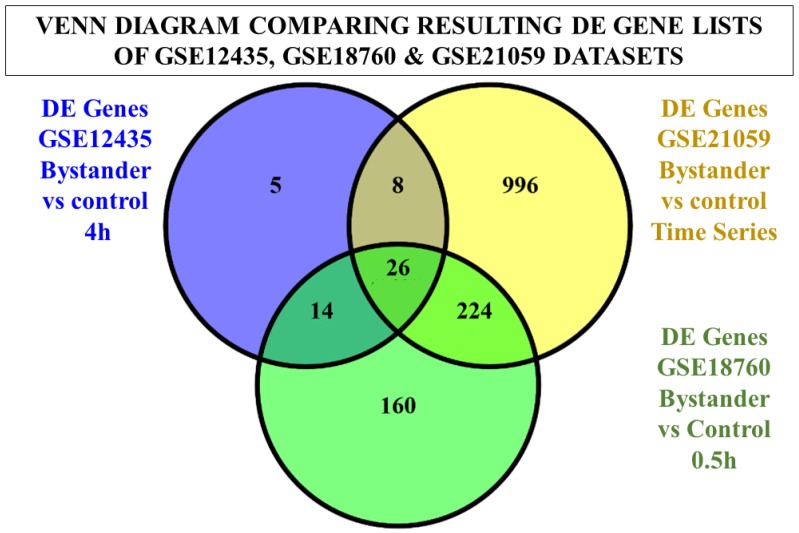
Venn diagram of DE genes lists regarding the GSE12435, GSE18760, and GSE21059 datasets for the comparisons of bystander vs. control samples. The comparison resulted in 26 common DE genes.

Briefly, in four out of seven datasets, differentially expressed (DE) genes were identified from the comparison of Bystander vs. Control samples, whereas the Irradiated vs. Control comparison resulted in plenty of DE genes for all datasets. However, the analysis of three specific datasets (GSE55869, GSE32091, GSE25772), in which cancer and immortalized cell lines were used, did not result in any DE genes regarding the comparison of bystander vs. control samples. Moreover, the highest expression alteration results, regarding the aforementioned comparison, were identified in the dataset with carbon-ion irradiation. In addition, the GSE12435, GSE18760, and GSE21059 datasets share significant similarities with respect to their experimental protocol despite the fact that the same cell type, type of particles, dose of radiation, and experimental procedure were followed in those three datasets. An important difference regarding all datasets has to do with the various time points that have been used for the RNA extraction after irradiation. Thus, we compared the resulting DE gene lists of the comparisons of bystander vs. control samples, in order to investigate whether there are common genes with the same differential expression direction at identical time points. Firstly, we compared the DE gene lists as depicted in the Venn diagram of Figure 1, which resulted in 26 common DE genes shared by all three datasets (Figure S2), regarding the comparison of bystander vs. control samples. 

Furthermore, by comparing the expression values across the same time points of the aforementioned datasets, we found that the majority of DE genes had similar values. The common DE genes are represented in Table 2.

### 2.2. Functional Enrichment Analysis

In order to highlight common molecular mechanisms evoked by RIBE, we exploited the functional enrichment results from three different biomedical ontologies (Gene Ontology (GO)) [14,15], Reactome pathways [16,17], and Mouse Genome Informatics (MGI) [18,19,20]), as derived by the BioInfoMiner (BIM) [13] interpretation web platform, yielding overlapping semantic terms above a certain level, across transcriptomic datasets. More specifically, we identified biological processes that were found to be significantly overrepresented in at least three out of six DE lists, concerning Bystander and Irradiated samples vs. controls with α-particles IR and two out of four with carbon-ion IR (Table 3 and Table 4).

Firstly, as illustrated in Table 3 for GO and in the Supplementary material for MGI (Table S1) and Reactome (Table S5), common functional terms were derived with the aid of BIM concerning the microarray datasets with α-particles IR. Many of the observed terms are related to the response to metal ions, the inflammation response, and protein misfolding-related processes. Additionally, GO terms related to the regulation of the Wnt signalling pathway and to non-canonical NF-kappaB activation, have been detected.

Similarly, as is illustrated for GO (Table 4) and in the supplementary material (Table S2) for MGI and Reactome (Table S6), common functional terms through BIM were observed for different time-points in the case of carbon-ion IR. Among the obtained terms, there are pathways linked to the negative regulation of metabolic processes, cell migration, and motility. Interestingly, a number of functional terms specific to either α-particles or carbon-ion datasets were also derived.

Next, we aimed to extract the instrumental, functional processes emerging from the comparisons of bystander vs. control and irradiated vs. control samples, respectively, in order to delineate the molecular landscape of RIBE and host response upon direct irradiation. BioInfoMiner functional enrichment analysis was performed using, as the input, significant DE gene lists from the datasets GSE12435 and GSE18760 for the α-particles IR and GSE8993 for the carbon-ion IR, respectively. In addition to the enrichment analysis, we performed gene prioritization regarding the datasets GSE12435 and GSE18760 for the α-particles IR and GSE8993 for the carbon-ion IR.

By combining DE gene lists derived from either bystander vs. control or irradiated vs. control comparisons for each of the aforementioned datasets, we derived the respective unique DE gene lists. Then, we fused them in four consensus gene lists: two for α-particles and two for carbon-ions, respectively. Finally, we performed comparative enrichment analysis on these gene lists as shown in Table 5 and Table 6 (respectively for MGI Tables S3 and S4 and for Reactome Tables S7 and S8).

In addition, common as well as distinct biological processes and molecular pathways between directly irradiated and bystander cell responses + samples control were derived, in order to gain an overview of RIBE. In the case of α-particles IR, common biological processes for both bystander and irradiated cells included the response to metal ions, unfolded protein response, and activation of the Wnt signalling pathway. On the contrary, distinct biological mechanisms included cell chemotaxis, migration, the inflammatory response, and the response to wounding, which were only found in bystander DE genes, whereas biological processes such as the DNA damage response, regulation of the mitotic cell cycle, and the apoptotic process were only detected in irradiated ones (Table 5). 

Similarly, common mechanisms have been found in the case of carbon-ion IR between bystander-irradiated cells with the most prevalent ones being, the regulation of cell migration, the RNA metabolic process, and the biosynthetic process. Unique biological processes of bystander cells are related to the regulation of the release of cytochrome from mitochondria, the regulation of oxidative phosphorylation and excretion, and the response to oxygen levels. Lastly, cell cycle arrest, and the regulation of cell migration, the *p38MAPK* cascade, *mTOR* signalling, and the extrinsic apoptotic signalling pathway were unique molecular processes observed in irradiated cells with carbon ion IR (Table 6).

Finally, from all the resulting DE gene lists of the datasets GSE18760, GSE12435, GSE21059, and GSE8993 for the bystander vs. control comparisons, 11 genes were common in at least three out of four datasets. These genes are presented in Table 7. Some of them were also derived from BIM as pivotal linker genes, cross-regulating diverse cellular processes. These genes can be identified as key-players underlying the functional pattern of bystander effects. Genes like *IL1A* and *IL1B* encode cytokines, which induce inflammatory and immune responses [21,22,23]. *CXCL8* and *CXCL2* are genes encoding secreted proteins of the chemokine superfamily mediators of the inflammatory response [24,25]. *FGF2* is a growth factor implicated in various biological processes such as wound healing, tumour growth, and angiogenesis [26,27]. *PTGS2* is a Prostaglandin-endoperoxide synthase involved in inflammation and mitogenesis [28,29]. *TNFAIP3* is involved in immune and inflammatory responses mediated by cytokines [30,31]. Lastly, *NFKBIZ* is known to play a crucial role in the modulation of inflammatory responses [32,33].

### 2.3. Rank Aggregation of Linker Genes

**Figure 2:**
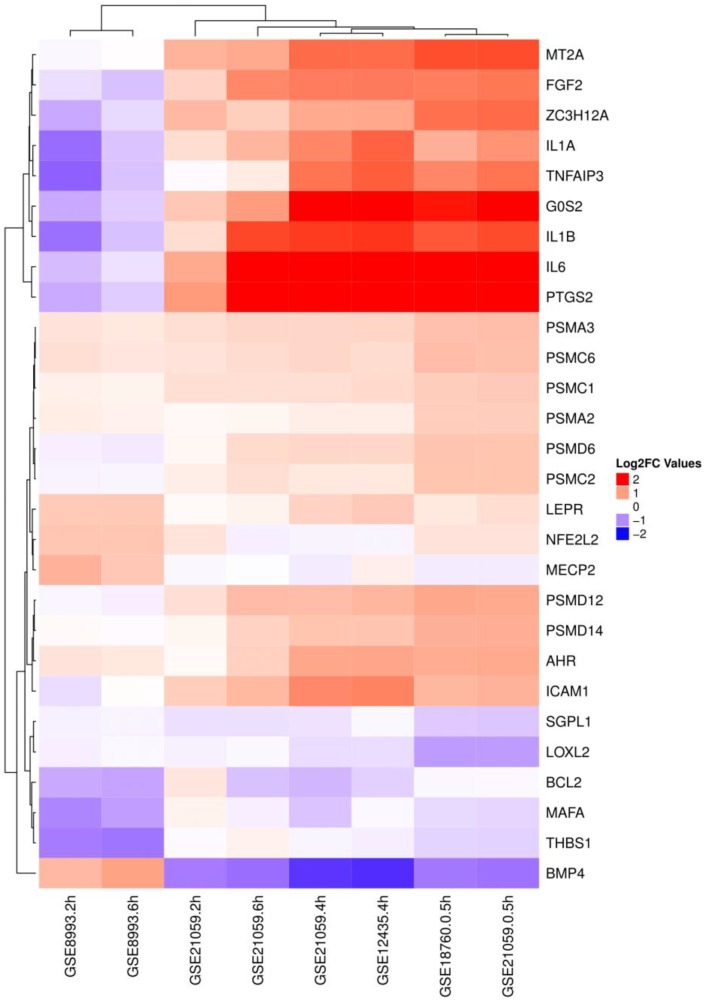
Heat map of the RIBE gene signature regarding the GSE12435*, GSE18760*, GSE21059*, and GSE8993^+^ datasets for the comparisons of bystander vs. control samples (GSEs with an asterisk highlight α-particles IR whereas the one marked with the plus symbol underlines carbon-ion IR). The relative Log2FC samples are represented in a ternary color format with red signifying: upregulation, blue: down regulation, and white: no alteration of gene expression regarding the controls.

In order to identify putative instrumental gene signatures of RIBE, we performed gene prioritization using BIM with different vocabularies (GO, Reactome Pathways and MGI), regardless of the time point or IR type. From the three resulting prioritized gene lists s for each bystander vs. control dataset comparison (GSE12435, GSE18760, GSE21059, and GSE8993 for 2 h and for 6 h) we performed rank aggregation, a method suitable for the optimal sorting of composite gene lists, (see Materials and Methods Section 4.2), which resulted in the following 28 ranked genes (Table 8):

In the next heat map (Figure 2), the relative log2FC of each of the pivotal genes is shown, comprising the RIBE signature set from the above table in each comparison.

## 3. Discussion

In the current study, the application of an integrative workflow to seven RIBE-related microarray datasets deposited in GEO (GSE55869 [34], GSE32091 [35], GSE21059 [36], GSE25772 [37], GSE18760 [38], GSE12435 [39], GSE8993 [40]), led to interesting findings regarding the underlying molecular mechanisms. 

Through rigorous standardized normalization and statistical selection, functional enrichment analysis, and gene prioritization based on functional mapping to various gene annotation vocabularies (GO, MGI, Reactome), we managed to overcome confounding factors and discrepancies resulting from major differences in the experimental design (various irradiation doses, several cell lines, and diverse types of IR). Ultimately, we identified specific conserved molecular pathways and mechanisms concerning the responses of bystander human cells to IR. 

More specifically, the highlighted molecular mechanisms include processes instrumental for the manifestation and modulation of the inflammatory response, aberrant wound healing, and tumorigenicity, like the activation of NF-kappaB in B cells, G1/S DNA Damage Check points, the activation of matrix metalloproteinases, the stabilization of p53, Wnt signalling, extracellular matrix organization, the regulation of apoptosis, and non-canonical NF-kB signalling (Figure S3). 

Regarding now, the GSE55869 dataset (H1299 cell line, non-small cell lung carcinoma, irradiated with α-particles), differential expression was observed only in the case of the comparison between irradiated vs. control samples (Figure S1). As expected, based on the subsequent functional enrichment analysis, this small subset is mainly linked to biological processes implicated in cell growth and proliferation (mitotic cell cycle process, cell division, chromosome segregation, and sister chromatid cohesion). Moreover, the vast majority of genes that were annotated to the above biological mechanisms were down-regulated, something which supports the direct cytostatic effect of IR in cancer cell lines [41]. The difference in the extent of the response observed is probably attributed to the priming through epigenetic reprogramming that cancer cells have undergone during their carcinogenic evolution, which renders them resistant to IR exposure. Their immortalization, a result of the aberrant activation of DDR, is largely facilitated by the inflammatory signaling mechanism, which is constitutively integrated as a key module for the carcinogenic transformation. The fact that in the case of bystander cancer cells, no significant genes arise through the comparison, should be seen rather than a small, sample-size issue, as a finding, which seems to preclude the availability of further, distinct inflammatory mechanisms, than those already observed. As the scope in this study is mostly to dissect the fundamental molecular mechanisms of the cell response to IR, and not emphasize the applied therapeutic aspect of treating cells with IR, the utilization of healthy stem cells, namely fibroblasts, with an intact inflammatory response mechanism, enables the detailed observation of a broader profile of molecular actions than those observed in cell models with aberrant response mechanisms.

Another important observation concerns the distinct biological profile of the RIBE response, regarding the different modes of IR (particles used for the irradiation of the cells). In particular, our results suggest different molecular mechanisms of host response to irradiation with α-particles than to irradiation with carbon-ion, with the difference being type-, but also possibly, dose-related. As shown in Table 8, many genes, albeit found as DE in both conditions, presented a different direction of gene expression alteration (upregulated in α-particles and down-regulated in carbon-ions). This opposite effect is further supported by the results of the functional enrichment analysis. In the case of α-particles, biological processes implicated in the inflammatory response, wound healing, cell proliferation, and cell migration were enriched, whereas in carbon-ion mechanisms, such as the regulation of cell death, the response to TNF, hypoxia, heat, and interleukins take the lead. The above findings apparently indicate that bystander cells responding to the irradiation of cells with α-particles are able to mobilize mostly survival functions, coping efficiently with the stress they undergo, unlike bystander cells responding to carbon-ion IR, which mostly converge to apoptotic death.

Moreover, the gene prioritization approach performed above enabled the inference of a small number of candidate genes that might play a pivotal role in the manifestation of RIBE. In particular, eleven DE genes were identified as common from the five “bystander” DE gene lists. From these genes, two cytokines (*IL1A, IL1B*) and the cyclooxygenase-2 (*PTGS2*) were identified as linker genes through BioInfoMiner, participating in a broad spectrum of diverse cellular processes, in the majority of the datasets. These specific genes have also been reported in previous studies to be associated with the progression of RIBE, mainly through the orchestration of immune and inflammatory responses and crosstalk [37,38,39]. In parallel, the rest of the common genes such as *SAT1, TNFAIP3, CXCL2*, and *FGF2*, were characterized as linker genes in at least one dataset. The latter are involved in immunoregulatory processes, polyamine metabolism [42,43,44], the inhibition of NF-kappa B [30], proliferation, and wound healing [45].

**Figure 3:**
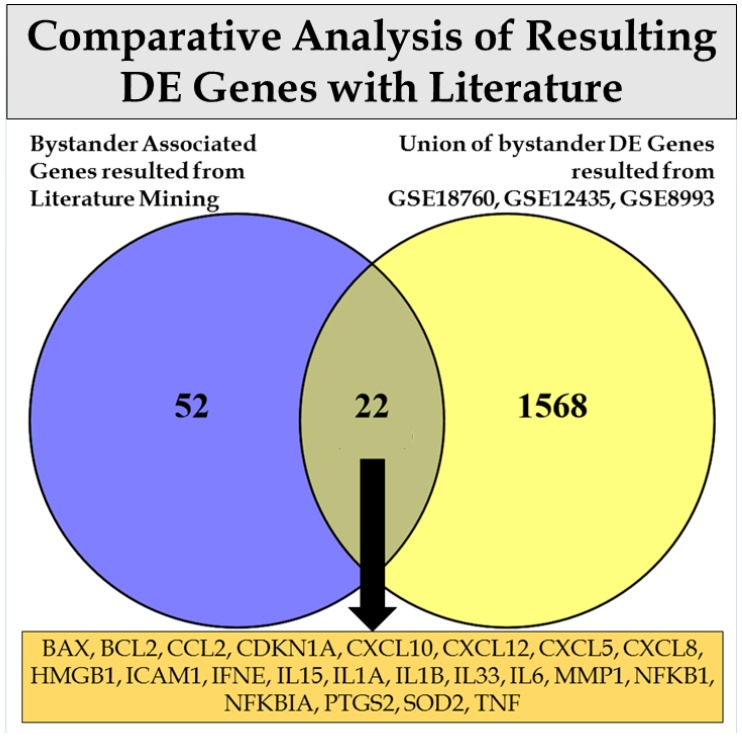
Venn diagram comparing a gene list associated with bystander effects derived from literature mining from the study of Nikitaki et al. [46] and a union of DE genes resulting from the statistical analysis of the GSE18760, GSE12435, and GSE8993 datasets for the comparisons of bystander vs. control samples. The comparison resulted in 22 common genes.

In parallel, we further explored the validity of one of the aforementioned derived DE gene lists, particularly the one formed from the union of bystander comparisons from the GSE18760, GSE12435, and GSE8993 datasets, with a reference literature-mined gene list regarding RIBE, proposed by the study of Nikitaki et al. [46]. From this comparison, 22 from the 74 genes were identified as common, mostly including interleukins, chemokines, and genes associated with apoptosis (Figure 3).

Finally, in order to derive a more compact and robust gene signature holistically describing RIBE, we performed functional enrichment analysis and gene prioritization exploiting different hierarchical biological vocabularies (GO, MGI, Reactome), with the aim to identify linker genes for diverse scopes in cellular physiology. Starting from the results of BIM gene prioritization for different vocabularies and using them as an input for the R package RankAggreg, a final subset of 28 pivotal genes was derived, representing candidate key-players for RIBE. The robustness of our methodology in this step is not solely limited to the gene expression, but through the utilization of different biological vocabularies, to the topological properties of the semantic networks delineated, describing the functional involvement of each gene, thus robustly promoting genes with a high regulatory impact in diverse cellular processes, representing functional proxies of their mode of operation. This is further illustrated in Table 9.

In this direction, both GO and MGI -ranked gene lists pinpoint common genes, including IL1-B, IL-1A, IL6, and PTGS2, with strongly established, immunoregulatory and inflammatory effects. On the other hand, there are also some significantly altered genes traced due to the use of MGI, such as MECP2, which is implicated in DNA methylation [47], as well as SGPL1 and GOS2 genes, mainly related to lipid metabolism [48,49]. Moreover, the Reactome pathway database yields the most distinct biological subset of linker genes, in comparison to GO and MGI, highlighting genes participating in the composition of the proteasome complex/component (PSMD6, PSMA2, PSMC1, etc.). Interestingly, it has been demonstrated in previous published studies that the proteasome has a primary role in the regulation of responses to IR [50,51], oxidative stress [52,53], and the regulation of apoptosis [54,55]. Overall, the final consensus signature comprises genes assuring the cross-talk among a diverse spectrum of distinct biological processes, which altogether could be considered as hallmarks of RIBE.

## 4. Materials and Methods 

### 4.1. Data Acquisition

Raw data comprised various microarray datasets, obtained from the public repository Gene Expression Omnibus. Specific microarray datasets were selected from the public repository GEO, using the term “radiation bystander effect”. From the total 10 results with human cell lines, seven microarray datasets related to RIBE (GSE55869 [34], GSE32091 [35], GSE21059 [36], GSE25772 [37], GSE18760 [38], GSE12435 [39], GSE8993 [40]) have been used for the analysis. The remaining three datasets have been excluded for reasons of inconsistency between files of sample and data relationship format and the different purpose of the experiment. Details and experimental design information of each dataset are illustrated in the following table (Table 10).

In GSE12435, GSE18760, and GSE21059, α-particles were used for the irradiation of the cells with a 0.5 Gray irradiation dose in the IMR-90 primary lung fibroblasts cell line. For the microarray experiment, the Agilent-014850 whole human genome microarray 4x44K, GPL6480 platform was used.

In GSE12435, the total RNA was isolated after four hours from the irradiation of the cells. The dataset contains four control (sham-irradiated) biological replicates, four irradiated biological replicates, and four bystander biological replicates.

In GSE18760, the total RNA was isolated after 30 min from the irradiation of the cells. The dataset contains four control (sham-irradiated) biological replicates, four irradiated biological replicates, and four bystander biological replicates.

In GSE21059, the total RNA was isolated at several time points (30 min, and 1, 2, 4, 6, and 24 h) from the irradiation of the cells. The dataset contains four control (sham-irradiated) biological replicates per time-point (26 samples), four irradiated biological replicates per time-point (26 samples), and four bystander biological replicates per time point (26 samples).

In GSE55869, α-particles were used for the irradiation of the cells with a 1 Gray irradiation dose in the H1299 non-small cell lung carcinoma cell line. For the microarray experiment, the Agilent-026652, Whole Genome, Human Microarray 4x44K v2, GPL13497 platform was used (Agilent Technologies, St. Clara, CA, USA). The total RNA was isolated after four hours from the irradiation of the cells. The dataset contains five control (non-sham-irradiated) biological replicates, five irradiated biological replicates, five controls of irradiated biological replicates, five bystander biological replicates, five controls of bystander biological replicates, and the same samples with shRAD9 cells. For this study, the samples of shRAD9 have been excluded.

In GSE3201, α-particles were used for the irradiation of the cells with a 0.1 Gray irradiation dose in the F11-hTERT immortalized foreskin fibroblasts cell line. For the microarray experiment, the Illumina HumanWG-6 v3.0 expression bead chip, GPL6884 platform was used (Illumina, San Diego, CA, USA). The total RNA was isolated after 4, 8, and 26 h from the irradiation of the cells. The dataset contains four control (sham-irradiated) biological replicates per time-point (12 samples), four irradiated biological replicates per time-point (12 samples), and four bystander biological replicates per time-point (12 samples).

In GSE25772, γ-rays were used for the irradiation of the cells with a dose of 2 Gy in the F11-hTERT immortalized foreskin fibroblasts cell line. For the microarray experiment, the Illumina HumanWG-6 v3.0 expression bead chip, GPL6884 platform was used. The total RNA was isolated after 4, 8, and 26 h from the irradiation of the cells. The dataset contains four control (sham-irradiated) biological replicates per time-point (12 samples), four irradiated biological replicates per time-point (12 samples), and four bystander biological replicates per time-point (12 samples).

In GSE8993, carbon-ions were used for the broad irradiation of the cells with 1.3, 0.13, and 0.013 Gy, and for micro-irradiation of the cells, with 0.12 Gy in the AG01522D primary normal human diploid skin fibroblasts cell line. For the microarray experiment, Agilent-014850 whole human genome microarray 4x44K, GPL6480 platform was used. The total RNA was isolated after 2 and 6 h from the irradiation of the cells. The dataset contains control (non-sham-irradiated) technical replicates for (micro-beam) bystander and (broad-beam) irradiated samples (four samples), two control (sham-irradiated) technical replicates for (micro-beam) bystander and (broad-beam) irradiated per time-point samples (eight samples), two bystander technical replicates per time-point, per irradiation dose (12 samples), and two irradiated technical replicates per time-point, per irradiation dose (12 samples).

Additionally, different experimental approaches were performed concerning the manifestation of the RIBE. In particular, three different experimental designs had been applied:

- Regarding the datasets GSE12435, GSE18760, GSE55869, GSE3201, and GSE21059, a method of the inner-outer dish was used, with the outer dish having a 6-micron Mylar strip base for the formation of the irradiated cells and the inner dish having one of 38-micron Mylar strips (which shields the cell from the IR) for the formation of the bystander cells [35,39].
- Regarding the dataset GSE25772, another experimental design was used, with the transference of conditioned medium from the irradiated cells to the “bystander” cells [37].
- Lastly, in the dataset GSE8993, micro beam and broad beam irradiation was used so as to form bystander and irradiated cells, respectively [40].

### 4.2. Computational Pipeline and Data Analysis

**Figure 4:**
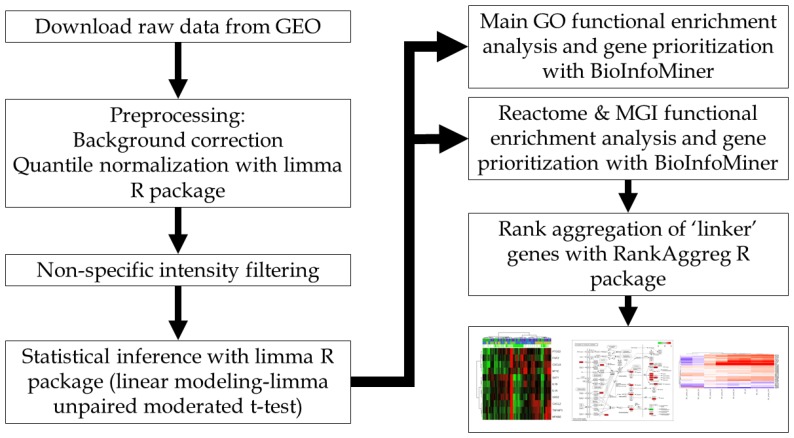
Computational pipeline of bioinformatic analysis.

For each dataset, raw data were acquired using the Bioconductor package GEOquery [56] and a pre-processing workflow for complete microarray analysis was implemented with R [R version 3.3.2 (31 October 2016)]/Bioconductor software [57,58] (Figure 4). For background correction [59] and quantile normalization [60], the limma [61,62,63] R package was used for both Agilent and Illumina platforms. Next, a non-specific intensity filtering procedure was applied, in order to remove low-expressed probesets in each dataset, based on probeset intensity distributions. In Illumina platform datasets, we used a further filtering step, based on a re-annotation pipeline regarding Illumina probe sequences quality information from the R package illuminaHumanv3.db [64]. The filtering procedure is described in detail in the limma user’s guide (Section 17.4) [65]. In parallel, exploratory analysis methodologies, such as unsupervised clustering, were applied to assess any quality problems and also to inspect putative batch effects regarding the experimental design. Finally, to measure the global expression alteration patterns between either bystander versus control or irradiated versus control samples, the moderated *t*-test (from limma R package) was applied, while batch/study information variable was included as a covariate factor in the linear model. For all statistical comparisons (except the ANOVA tests in some specific cases), we used the same double cutoff to obtain the DE gene lists: an absolute value of log_2_ fold change greater than 0.5 and an adjusted *p*-value less than 0.05 (FDR) [66].

The molecular pathway and functional analysis was performed using BioInfoMiner [13,67], which exploits several vocabularies with a hierarchical structure, such as Gene Ontology, Reactome Pathways, and MGI and HPO phenotype ontologies, in order to provide a multi-faceted, functional, gene-level description of the phenotypes studied. The analysis comprises the ranking and prioritization of enriched biological processes and genes.

We used BioInfoMiner as the basic tool in order to identify overrepresented functional terms, as well as to highlight subsets of genes with pivotal roles in orchestrating RIBE. Briefly, BioInfoMiner derives a subset of the input genes, in which the genes are ranked according to their functional association with multiple, distinct cellular processes. These subsets of genes, termed “linker genes”, are implicated as central actors in various distinct biological processes, thus providing a holistic view of the disease under investigation. The methodology is described in Koutsandreas et al. [67].

In order to derive a gene signature characterizing RIBE, we combined different subsets of linker genes, derived from the application of the methodology with different vocabularies, namely GO [14,15], Reactome [16,17], and MGI [18,19,20]. Firstly, we performed functional enrichment analysis and gene prioritization for every gene list of the aforementioned bystander comparisons, resulting in five linker gene lists for GO: five for Reactome and five for MGI vocabularies. Secondly, we performed rank aggregation of the linker gene ordered lists with the package R RankAggreg [68] for each vocabulary, resulting in three ranked linker gene lists. Finally, the union of these three gene lists resulted in 28 unique linker genes. The Venny [69] web tool was used for the illustration of Venn diagrams. For KEGG [70] pathway enrichment analysis we used Enrichr [71,72] and for the illustration of the derived enriched pathways we used Pathview [73,74] (supplementary material).

## 5. Conclusions

Through the implementation of a robust integrative bioinformatics analysis of transcriptomic data regarding the molecular investigation of RIBE, a consensus signature of 28 linker genes was derived (including *IL1-B, IL-1A, IL6*, and *PTGS2* with a pivotal role), which are associated with multiple and diverse underlying biological mechanisms. Interestingly, reverse gene expression was observed for a specific subset of DE genes, common in both α-particles and carbon-ion IR comparisons regarding RIBE, a finding that potentially suggests an alternate biological response mechanism adjustable to different modes of radiation. This is further supported by the functional enrichment results of the comparative analysis, highlighting distinct biological processes, such as induction of the inflammatory response, cell growth, and healing in bystander cells of α-particles IR experiments, whereas the positive regulation of apoptotic cell death is mainly affected in the case of carbon-ion IR. Overall, our results provide a detailed account for the molecular mechanisms implicated in RIBE, with potential interest in cancer therapeutics research. In this direction, our derived RIBE signature of candidate genes could be further investigated in other independent cancer transcriptomic datasets, in order to examine potentially interesting association patterns with cell survival and response to irradiation.

## Figures and Tables

**Table 1:** Numbers of differentially expressed genes (DE) resulting from statistical testing using False Discovery Rate (FDR) < 0.05 and |log2 Fold Change| > 0.5. Numbers in parentheses define the time that had passed after the irradiation for the isolation of the RNA from cells.

Dataset	GSE12435	GSE18760	GSE21059	GSE55869	GSE32091	GSE25772	GSE8993
Type of Radiation	α-particles					γ-rays	carbon-ions
DE Bystander vs. Control	53 (4 h)	424 (0.5 h)	1254 (ANOVA-time-series)	0	0	0	1003 (2 h) 796 (6 h)
DE Irradiated vs. Control	76 (4 h)	481 (0.5 h)	2399 (ANOVA-time-series)	47 (4 h)	3 (4 h) 0 (8 h) 0 (26 h)	271 (4 h) 223 (8 h) 1977 (26 h)	1502 (2 h) 1897 (6 h)
DE Common	39	339	1169	-	-	-	264 (2 h) 324 (6 h)

**Table 2:** The expression alterations of the 26 common DE genes. Values represent expression fold changes of bystander vs. control cells, on a Log2 scale. Values with bold and bold/italics illustrate similarity between the same time points of different datasets.

Common DE Genes	Fold Change in Expression							
Datasets	GSE18760	GSE12435	GSE21059					
**Time Points**	0.5 h	4 h	0.5 h	1 h	2 h	4 h	6 h	24 h
*MT1B*	*2.421*	*1.905*	*2.456*	0.898	1.122	*1.927*	1.244	1.185
*MT1E*	*2.574*	*2.165*	*2.620*	0.964	1.143	*2.178*	1.209	1.114
*MT1H*	*2.380*	*2.001*	*2.424*	0.982	1.076	*2.028*	1.186	1.205
*MT1X*	*2.528*	*2.002*	*2.480*	1.013	1.048	*2.033*	1.173	1.196
*MT2A*	*1.690*	*1.450*	*1.704*	0.678	0.790	*1.455*	0.885	0.975
*PTGS2*	*2.615*	*2.401*	*2.769*	0.842	1.036	*2.259*	2.616	0.323
*CXCL5*	*1.589*	*2.063*	*1.975*	0.383	0.133	*1.772*	2.335	1.154
*MMP3*	*2.582*	*1.932*	*2.690*	1.143	0.963	*1.901*	3.335	2.023
*MT1L*	*2.364*	*1.931*	*2.404*	0.898	1.014	*1.958*	1.192	1.280
*ARC*	*2.102*	*1.904*	*2.778*	0.603	−0.374	*1.289*	1.244	0.163
*TSLP*	*0.618*	*1.407*	*0.703*	0.628	0.466	*1.354*	0.829	1.043
*CXCL1*	*1.518*	*1.420*	*1.508*	0.673	0.761	*1.453*	1.160	0.836
*GPR68*	*0.824*	*1.709*	*0.893*	0.690	0.810	*1.707*	2.082	1.441
*MMP1*	*2.154*	*1.648*	*2.187*	1.078	0.941	*1.662*	2.827	1.366
*MMP10*	*1.098*	*1.666*	*1.262*	0.726	0.699	*1.549*	1.663	0.892
*KYNU*	*1.963*	*1.806*	*2.121*	1.220	0.876	*1.622*	1.385	1.332
*SLC16A6*	*1.723*	*1.709*	*1.888*	0.796	0.839	*1.579*	2.431	1.493
*SLC7A11*	*1.445*	*1.259*	*1.522*	1.076	0.946	*1.224*	0.887	1.033
*NAMPT*	*1.393*	*1.486*	*1.426*	0.659	0.524	*1.571*	0.736	0.639
*HSD11B1*	*1.509*	*1.500*	*1.620*	0.718	0.607	*1.442*	1.491	1.074
*LAMB3*	*1.548*	*1.443*	*1.702*	0.644	0.564	*1.383*	1.580	1.153
*PLA2G4A*	*1.115*	*1.199*	*1.229*	0.665	0.468	*1.138*	0.881	0.724
*C8orf4*	*1.277*	*1.486*	*1.353*	0.734	0.586	*1.432*	0.780	1.036
*EPHA4*	−*0.881*	−*1.109*	−*0.893*	−0.937	−0.727	−*0.704*	−0.628	−0.947
*ADGRG1*	*1.022*	*0.873*	*1.086*	0.540	0.131	*0.841*	0.548	1.123
*CCK*	*1.048*	*1.065*	*1.208*	0.570	0.273	*0.995*	0.869	0.867

**Table 3:** Common Gene Ontology (GO) terms resulting from functional enrichment analysis for bystander vs. control and irradiated vs. control comparisons of datasets with α-particles irradiation. Enrichment scores are given as a fraction value.

Gene Ontology	Datasets/Enrichments					
	GSE12435		GSE18760		GSE21059	
	Bystander 4 h	Irradiated 4 h	Bystander 0.5 h	Irradiated 0.5 h	Bystander Time-Series	Irradiated Time-Series
Cellular Response to zinc ion	5/18	6/18	9/18	9/18	10/18	11/18
Response to Zinc Ion	5/53	6/53	11/53	12/53	14/53	16/53
Cellular Response to Cadmium Ion	3/15	4/15	6/15	6/15	7/15	8/15
Cellular Response to Metal Ion	5/126	8/126	15/126	16/126	23/126	29/126
Response to Inorganic Substance	10/428	12/428	33/428	34/428	54/428	-
Cellular Response to Inorganic Substance	6/146	9/146	16/146	17/146	25/146	-
Response to Metal Ion	8/298	11/298	26/298	27/298	41/298	-
Protein Folding	-	-	-	17/211	34/211	54/211
Cytokine-Mediated Signalling Pathway	8/440	-	31/440	32/440	-	-
Regulation of NF-kappaB Import into Nucleus	3/44	-	7/44	7/44	-	-
Positive Regulation of Reactive Oxygen Species Biosynthetic Process	3/46	-	7/46	7/46	-	-
Cytokine-mediated Signalling Pathway	8/440	-	31/440	32/440	-	-
Regulation of Anatomical Structure Morphogenesis	-	-	57/934	56/934	105/934	163/934
Extracellular Matrix Disassembly	4/73	-	-	-	15/73	21/73
Embryonic Skeletal System Development	-	-	-	-	10/43	14/43
Regulation of Protein Modification Process	-	-	79/1616	-	155/1616	279/1616
Response to Unfolded Protein	-	-	7/45	8/45	10/45	15/45
Wnt Signalling Pathway, Planar Cell Polarity Pathway	-	-	11/99	11/99	17/99	26/99

**Table 4:** Common Gene Ontology terms resulting from functional enrichment analysis for bystander vs. control and irradiated vs. control comparisons of dataset GSE8993 with carbon-ion irradiation. Enrichment scores are given as a fraction value.

Gene Ontology	Dataset/Enrichments			
	GSE8993			
	Bystander 2 h	Irradiated 2 h	Bystander 6 h	Irradiated 6 h
Negative Regulation of Nucleobase-containing Compound Metabolic Process	112/1310	-	84/1310	188/1310
Negative Regulation of Cellular Biosynthetic Process	117/1394	-	88/1394	196/1394
Negative Regulation of Nitrogen Compound Metabolic Process	119/1425	-	90/1425	202/1425
Negative Regulation of RNA Metabolic Process	99/1178	-	79/1178	170/1178
Regulation of Cell Migration	62/662	91/662	-	113/662
Regulation of Epithelial Cell Migration	20/165	27/165	-	34/165
Negative Regulation of Cell Migration	-	34/206	19/206	39/206
Negative Regulation of Cellular Component Movement	-	39/247	22/247	44/247
Negative Regulation of Cell Motility	-	-	20/218	39/218

**Table 5:** Evaluation of differences in Gene Ontology terms resulting from functional enrichment analysis of datasets GSE12435 and GSE18760 from unique DE genes between comparisons of bystander vs. control and irradiated vs. control samples.

Unique Gene Ontology Terms α-Particles IR (GSE12435, GSE18760)	
Bystander	Irradiated
positive regulation of vasoconstriction	DNA damage response, signal transduction by p53 class mediator resulting in cell cycle arrest
polyamine catabolic process	activation of cysteine-type endopeptidase activity involved in apoptotic signalling pathway
cell chemotaxis	extrinsic apoptotic signalling pathway via death domain receptors
regulation of response to external stimulus	negative regulation of G1/S transition of mitotic cell cycle
cell migration	regulation of apoptotic process
inflammatory response	nucleic acid phosphodiester bond hydrolysis
regulation of defence response to virus by host	activation of MAPKKK activity
regulation of response to wounding	atrioventricular valve morphogenesis
positive regulation of leukocyte migration	atrial septum development
positive regulation of cell-matrix adhesion	embryo development

**Table 6:** Evaluation of differences in Gene Ontology terms resulting from functional enrichment analysis of datasets GSE8993 from unique DE genes between comparisons of bystander vs. control and irradiated vs. control samples.

Unique Gene Ontology Terms Carbon-Ion IR (GSE8993)			
Bystander	Enrichment	Irradiated	Enrichment
positive regulation of mitochondrial outer membrane permeabilization involved in apoptotic signalling pathway	9/35	positive regulation of protein binding	24/75
positive regulation of protein homooligomerization	4/8	cell cycle arrest	34/148
negative regulation of intracellular protein transport	13/84	cellular component disassembly involved in execution phase of apoptosis	10/25
positive regulation of release of cytochrome c from mitochondria	7/28	cellular response to transforming growth factor β stimulus	16/53
regulation of oxidative phosphorylation	5/15	regulation of cell migration	123/662
regulation of steroid hormone secretion	5/19	response to transforming growth factor β	17/59
mitochondrial membrane organization	12/90	regulation of p38MAPK cascade	10/26
cellular response to oxygen levels	14/111	regulation of TOR signalling	19/70
regulation of excretion	6/25	positive regulation of extrinsic apoptotic signalling pathway	15/52
multicellular organismal response to stress	9/59	regulation of cell-matrix adhesion	22/91

**Table 7:** Common DE genes resulting from all comparisons of bystander vs. control samples of the analyzed datasets. Expression values are presented as log2FC and values with * indicating genes suggested as linker genes by the GO functional enrichment analysis of BioInfoMiner.

Common Genes	Bystander					
	α-Particles				Carbon Ion	
	GSE18760	GSE12435	GSE21059		GSE8993	
	0.5 h	4 h	2 h	6 h	2 h	6 h
***IL1A***	0.81 *	1.53 *	0.34	0.76	−1.27	−0.5 *
***IL1B***	1.62 *	1.85 *	0.36	1.74	−1.23 *	−0.54 *
***NFKBIZ***	1.32	1.44	0.51	0.85	−1.41	−0.53
***SAT1***	1.16	0.91 *	-	0.4	0.52	0.54
***TNFAIP3***	1.22 *	1.58 *	-	0.22	−1.35	−0.52
***CXCL2***	2.42 *	2.64	0.64	1.14	−0.92	-
***G0S2***	1.96	2.15	0.57	1.02	−0.73	-
***MT1E***	2.57	2.16	1.1	1.2	−0.5	-
***PTGS2***	2.61 *	2.4 *	1.03 *	2.61 *	−0.73*	-
***CXCL8***	3.53 *	-	1.3	3.6	−1.36	−0.69
***FGF2***	1.29	1.31	-	-	-	−0.53 *

**Table 8:** Top ranked linker DE genes resulting from rank aggregation of each linker gene list vocabulary.

Ranked Linker DE Genes		
GO	MGI	Reactome Pathways
***IL6***	*PTGS2*	*PSMD6*
***ZC3H12A***	*BMP4*	*PSMA2*
***PTGS2***	*IL6*	*PSMA3*
***BCL2***	*LEPR*	*PSMD14*
***BMP4***	*IL1B*	*PSMC1*
***THBS1***	*NFE2L2*	*PSMC2*
***IL1A***	*AHR*	*PSMC6*
IL1B	MECP2	IL1B
***TNFAIP3***	*SGPL1*	*FGF2*
***ICAM1***	*G0S2*	*PSMD12*
***MT2A***		*LOXL2*
		*MAFA*

**Table 9:** Top 5 Ranked Linker Genes resulting from ranked aggregation from Linker gene lists for bystander vs. control comparisons of datasets GSE18760, GSE12435, and GSE8993. Top enriched clusters are illustrated for each Linker gene.

Top 5 Ranked Linker Genes GO	Enriched Clusters	Top 5 Ranked Linker Genes MGI	Enriched Clusters	Top 5 Ranked Linker Genes Reactome	Enriched Clusters
***IL6***	inflammatory response, cytokine-mediated signaling pathway, cellular response to oxidative stress	*PTGS2*	abnormal wound healing, increased IgA level, abnormal IgG3 level	*PSMD6*	Hedgehog “on” state, Degradation of beta-catenin by the destruction complex, Beta-catenin independent WNT signaling, PCP/CE pathway, Regulation of activated PAK-2p34 by proteasome mediated degradation, CLEC7A (Dectin-1) signaling, Metabolism of polyamines
***ZC3H12A***	negative regulation of cell death, cellular response to oxidative stress, inflammatory response, regulation of apoptotic process	*BMP4*	increased apoptosis	*PSMA2*	
***PTGS2***	cellular response to oxidative stress, cellular response to metal ion, cellular response to fluid shear stress, regulation of apoptotic process	*IL6*	increased IgA level, abnormal interferon-gamma secretion, abnormal circulating interleukin level	*PSMA3*	
***BCL2***	negative regulation of extrinsic apoptotic signaling pathway, response to hypoxia	*LEPR*	increased apoptosis, abnormal interferon-gamma secretion, abnormal circulating interleukin level	*PSMD14*	
**BMP4**	system development, positive regulation of cell migration, positive regulation of protein modification process	*IL1B*	abnormal wound healing, abnormal macrophage physiology, decreased interleukin-6 secretion	*PSMC1*	

**Table 10:** Information about microarray datasets used in the bioinformatic analysis.

GEO Accession Number	GSE18760	GSE12435	GSE21059	GSE55869	GSE32091	GSE25772	GSE8993
Type of Radiation	α-particles					γ-rays	carbon-ion
Time of Extraction of Total RNA after Irradiation (h)	0.5	4	0.5, 1, 2, 4, 6, 24	4	4, 8, 26		2, 6
Irradiation Dose (Gy)	0.5			1	0.1	2	1.3, 0.13, 0.013
Cell Line	IMR-90 primary lung fibroblasts			H1299 non-small cell lung carcinoma	F11-hTERT immortalized foreskin fibroblasts		AG01522D primary normal human diploid skin fibroblasts

## Supplementary Materials

The following are available online at www.mdpi.com/2072-6694/9/12/160/s1, Figure S1: Gene-Bar plot of the 16 GO terms from the comparison irradiated vs. control in the NSCLC dataset, Figure S2: Illustrative Heatmap of the 26 common DE genes, Figure S3: Illustrative example of NF-kappaB signaling pathway, Table S1: Common Mouse Genome Informatics (MGI) terms of α-particles IR, Table S2: Common MGI terms of carbon-ion IR, Table S3: Evaluation of differences in MGI terms of GSE12435 and GSE18760 datasets, Table S4: Evaluation of differences in MGI terms of GSE8993 dataset, Table S5: Common Reactome pathways terms of α-particles IR, TableS6: Common Reactome pathways terms of carbon-ion IR, Table S7: Evaluation of differences in Reactome pathways terms of GSE12435 and GSE18760 datasets, Table S8: Evaluation of differences in Reactome pathways terms of dataset GSE8993.
