# Supplementary material for: Integrative Bioinformatic Analysis of Transcriptomic Data Identifies Conserved Molecular Pathways Underlying Ionizing Radiation-Induced Bystander Effects (RIBE)
Source: Cancers (Basel). 2017 Nov 25;9(12):160. doi: 10.3390/cancers9120160 (PMC5742808; doi:10.3390/cancers9120160)
Supplement: Supplementary file 1 [file cancers-09-00160-s001.pdf]

Supplementary Materials: Integrative bioinformatic analysis of transcriptomic data identifies conserved molecular pathways underlying ionizing radiation-induced bystander effects (RIBE)

Constantinos Yeles, Efstathios-Iason Vlachavas, Olga Papadodima, Eleftherios Pilalis, Constantinos E. Vorgias, Alexandros G. Georgakilas, and Aristotelis Chatziioannou

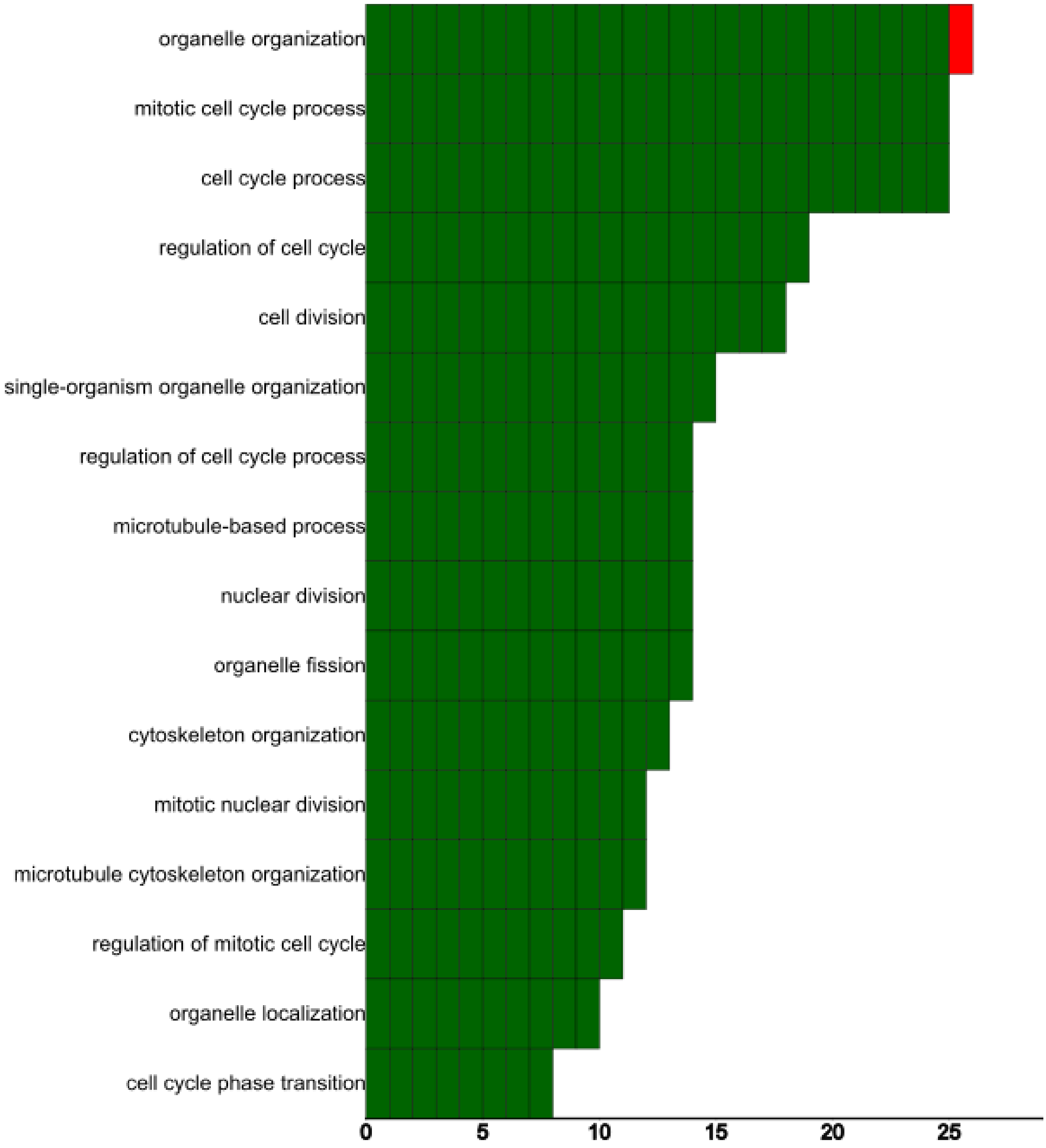

Figure S1. Bar plot of the top 16 Gene Ontology terms, resulting from functional enrichment analysis of gene list comparison irradiated vs control samples of GSE55869 with BioInfoMiner, ranked according to the number of associated genes. Green color illustrates the down-regulation and red the up-regulation of a gene. As it is represented the vast majority of the genes are down-regulated. The cell-line which used in GSE55869 was H1299 non-small cell lung carcinoma.

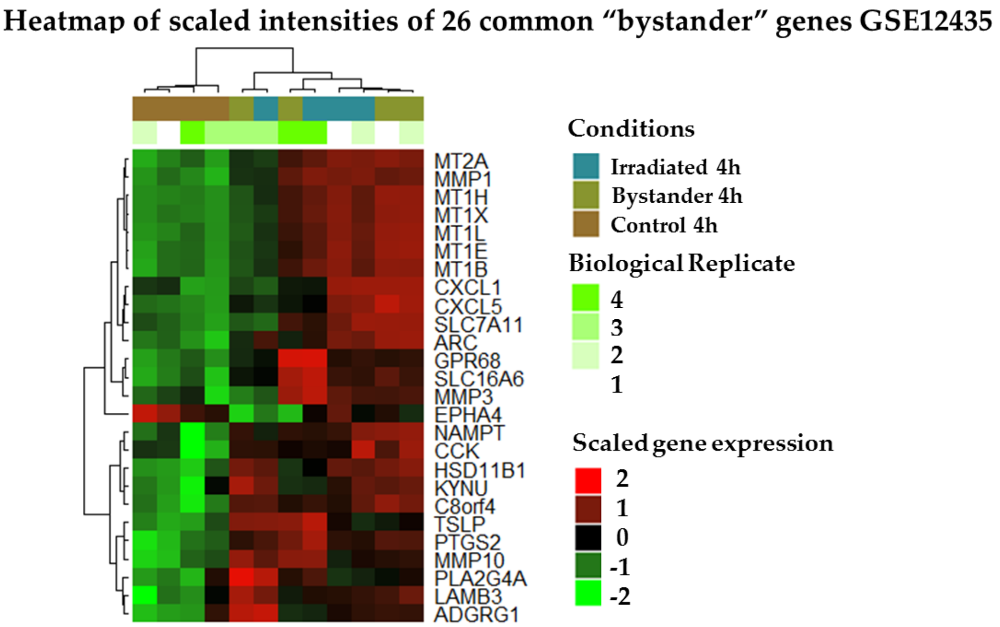

Figure S2. Heatmap of the 26 common DE genes resulting from comparing gene lists of GSE12435, GSE21059 and GSE18760 for the comparison bystander vs control samples. Hierarchical clustering method: ward, distance: Euclidean. The gene expressions have been scaled so as to have mean expression of zero and standard deviation of one.

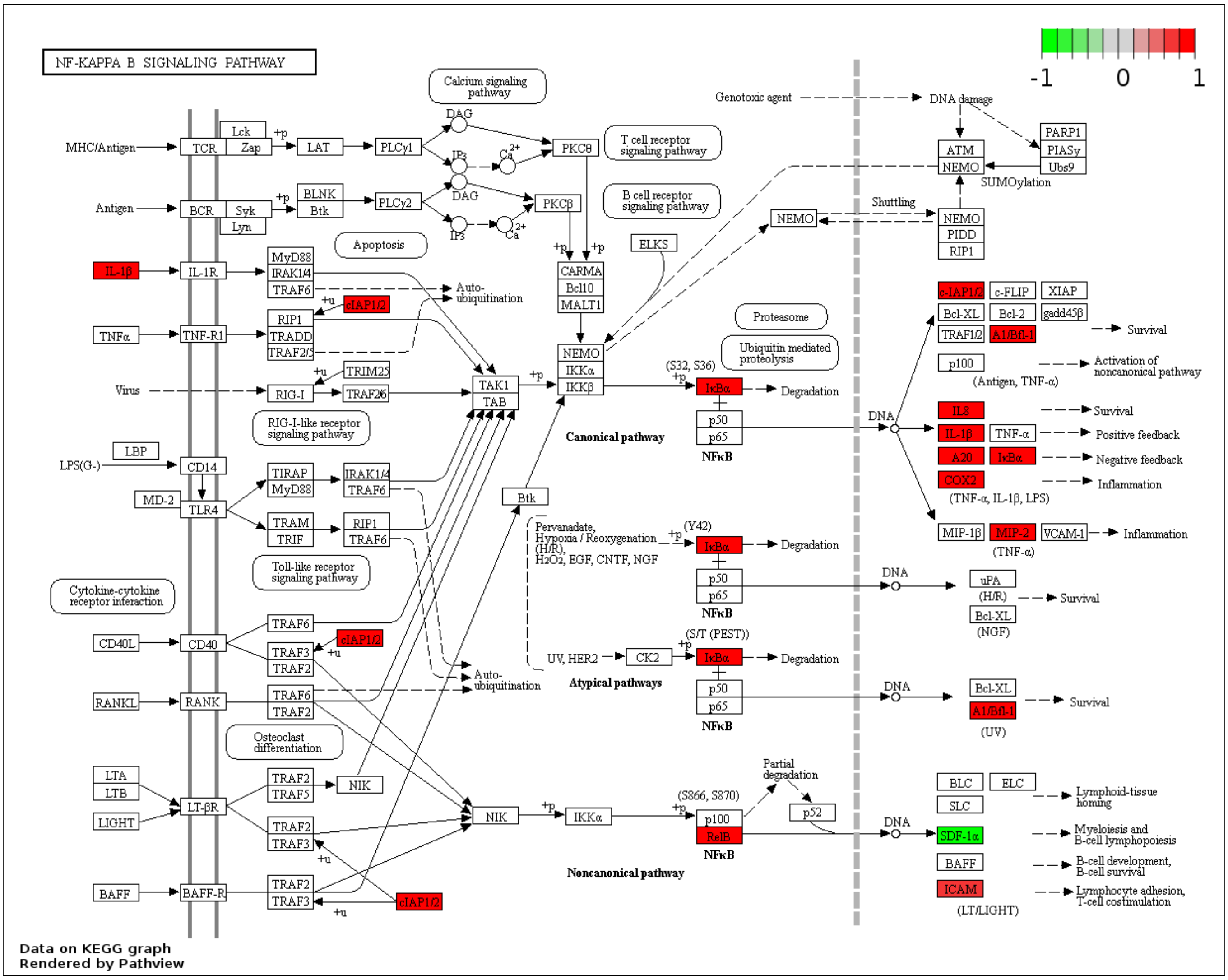

Figure S3. Illustrative example of NF-kappaB signaling pathway found significantly enriched after pathway analysis with Enrichr and illustrated with Pathview, using as an input the DE gene list from 0.5h Bystander versus control comparison.

Table S1. Common Mouse Genome Informatics (MGI) terms resulting from functional enrichment analysis for bystander vs control and irradiated vs control comparisons of datasets with a-particles irradiation. Enrichment scores are given as a fraction value.

MGI	Datasets / Enrichments					
	GSE12435		GSE18760		GSE21059	
	Bystander 4 h	Irradiated 4 h	Bystander 0.5 h	Irradiated 0.5 h	Bystander Time-series	Irradiated Time-series
**small kidney**	-	7/167	13/167	13/167	24/167	-
**abnormal macrophage physiology**	8/337	10/337	25/337	24/337	-	-
**abnormal cytokine secretion**	13/529	13/529	34/529	32/529	-	-
**abnormal incidence of induced tumors**	6/181	8/181	14/181	14/181	-	-

**increased fibroblast proliferation**	-	4/37	6/37	5/37	-	-
**increased sensitivity to induced morbidity/mortality**	-	8/274	-	19/274	-	-
**increased tumor growth/size**	-	-	7/38	7/38	8/38	-
**abnormal innate immunity**	10/503	-	32/503	30/503	-	-
**decreased interleukin-6 secretion**	4/78	-	8/78	8/78	-	-

Table S2. Common MGI terms resulted from functional enrichment analysis for bystander vs control and irradiated vs control comparisons of dataset GSE8993 with carbon-ion irradiation. Enrichment scores are given as a fraction value.

**MGI**	**Dataset / Enrichments**			
	**GSE8983**			
	**Bystander 2 h**	**Irradiated 2 h**	**Bystander 6 h**	**Irradiated 6 h**
**increased urine glucose level**	6/20	-	5/20	-
**neoplasm**	55/542	-	38/542	-
**abnormal kidney mesenchyme morphology**	6/28	-	5/28	-
**prenatal lethality**	-	220/1884	-	276/1884
**ventricular septal defect**	-	28/165	-	32/165
**embryonic growth arrest**	-	35/241	-	45/241

Table S3. Evaluation of differences in MGI terms resulting from functional enrichment analysis of datasets GSE12435 and GSE18760 from unique DE genes between comparisons bystander vs control and irradiated vs control.

**Unique MGI terms a-particles IR (GSE12435, GSE18760)**	
**Bystander**	**Irradiated**
enhanced wound healing	increased anti-histone antibody level
decreased susceptibility to type IV hypersensitivity reaction	increased anti-single stranded DNA antibody level
abnormal angiogenesis	decreased immature B cell number
abnormal thymus involution	abnormal T cell proliferation
increased hepatoma incidence	decreased mature B cell number
abnormal T cell physiology	increased T cell proliferation
prenatal lethality prior to heart atrial septation	decreased B-1 B cell number
abnormal chondrocyte morphology	increased inguinal fat pad weight

increased myeloid cell number in bone marrow	increased autoantibody level
osteoarthritis	decreased sensitivity to skin irradiation

Table S4. Evaluation of differences in MGI terms resulted from functional enrichment analysis of dataset GSE8993 from unique DE genes between comparisons bystander vs control and irradiated vs control.

Unique MGI terms carbon-ion IR (GSE8993)	
**Bystander**	**Irradiated**
peritoneal inflammation	heart inflammation
increased circulating tumor necrosis factor level	perinatal lethality, incomplete penetrance
decreased cytotoxic T cell cytolysis	dystrophic muscle
decreased double-positive T cell number	decreased fibroblast cell migration
increased circulating interleukin-17 level	abnormal vascular development
decreased T cell proliferation	skeletal muscle fiber necrosis
increased pre-B cell number	abnormal liver development

Table S5. Common Reactome pathways terms resulting from functional enrichment analysis for bystander vs control and irradiated vs control comparisons of datasets with a-particles irradiation. Enrichment scores are given as a fraction value.

Reactome pathways	Datasets / Enrichments					
	GSE12435		GSE18760		GSE21059	
	Bystander 4 h	Irradiated 4 h	Bystander 0.5 h	Irradiated 0.5 h	Bystander Time-series	Irradiated Time-series
**Stabilization of p53**	-	-	10/55	10/55	11/55	18/55
**G1/S DNA Damage Checkpoints**	-	-	10/66	10/66	11/66	19/66
**Hh mutants that don't undergo autocatalytic processing are degraded by ERAD**	-	-	9/56	9/56	10/56	16/56
**Cyclin E associated events during G1/S transition**	-	-	10/69	10/69	13/69	19/69
**Cyclin A:Cdk2-associated events at S phase entry**	-	-	10/70	10/70	13/70	20/70
**Crosslinking of collagen fibrils**	-	-	4/11	5/11	6/11	6/11
**PCP/CE pathway**	-	-	11/91	11/91	16/91	25/91
**TP53 Regulates Transcription of Death Receptors and Ligands**	-	3/12	-	-	-	6/12

**Extracellular matrix organization**	6/289	-	-	-	38/289	-

Table S6. Common Reactome pathways terms resulted from functional enrichment analysis for bystander vs control and irradiated vs control comparisons of dataset GSE8993 with carbon-ion irradiation. Enrichment scores are given as a fraction value.

Reactome pathways	Dataset / Enrichments			
	GSE8983			
	Bystander 2 h	Irradiated 2 h	Bystander 6 h	Irradiated 6 h
**Circadian Clock**	9/62	14/62	-	16/62
**BMAL1:CLOCK,NPAS2 activates circadian gene expression**	8/42	8/42	-	11/42
**Laminin interactions**	-	9/30	-	10/30
**Pre-NOTCH Expression and Processing**	-	9/45	-	12/45
**Activation, translocation and oligomerization of BAX**	2/2	-	2/2	-
**RHO GTPases Activate ROCKs**	4/17	-	3/17	-
**Signaling by ERBB4**	27/319	-	23/319	-

Table S7. Evaluation of differences in Reactome pathways terms resulted from functional enrichment analysis of datasets GSE12435 and GSE18760 from unique DE genes between comparisons bystander vs control and irradiated vs control.

Unique Reactome terms a-particles IR (GSE12435, GSE18760)	
**Bystander**	**Irradiated**
RHO GTPases activate PKNs	TP53 Regulates Transcription of Cell Death Genes
RHO GTPases Activate ROCKs	PI3K/AKT Signaling in Cancer
RHO GTPases activate PAKs	Downstream signaling events of B Cell Receptor (BCR)
O-linked glycosylation	Constitutive Signaling by AKT1 E17K in Cancer
Defective TBXAS1 causes Ghosal hematodiaphyseal dysplasia (GHDD)	Death Receptor Signalling
PRC2 methylates histones and DNA	Transcription from mitochondrial promoters
Synthesis and interconversion of nucleotide di- and triphosphates	Role of LAT2/NTAL/LAB on calcium mobilization
RMTs methylate histone arginines	AKT phosphorylates targets in the cytosol
Interleukin-7 signaling	TP53 Regulates Transcription of Death Receptors and Ligands
EPHA-mediated growth cone collapse	TP53 Regulates Transcription of Genes Involved in G1 Cell Cycle Arrest

Table S8. Evaluation of differences in Reactome pathways terms resulted from functional enrichment analysis of dataset GSE8993 from unique DE genes between comparisons bystander vs control and irradiated vs control.

**Unique Reactome terms carbon-ion IR (GSE8993)**	
**Bystander**	**Irradiated**
**BH3-only proteins associate with and inactivate anti-apoptotic BCL-2 members**	Laminin interactions
**Activation, translocation and oligomerization of BAX**	Circadian Clock
**Nef Mediated CD4 Down-regulation**	Pre-NOTCH Transcription and Translation
**Cytosolic sensors of pathogen-associated DNA**	PPARA activates gene expression
**Signaling by FGFR2**	BMAL1:CLOCK,NPAS2 activates circadian gene expression
**Fc epsilon receptor (FCERI) signaling**	Regulation of lipid metabolism by Peroxisome proliferator-activated receptor alpha (PPARalpha)
**CLEC7A/inflammasome pathway**	Signaling by TGF-beta Receptor Complex

© 2017 by the authors; licensee MDPI, Basel, Switzerland. This article is an open access article distributed under the terms and conditions of the Creative Commons by Attribution (CC-BY) license (http://creativecommons.org/licenses/by/4.0/)
